# Chemically-Induced Cancers Do Not Originate from Bone Marrow-Derived Cells

**DOI:** 10.1371/journal.pone.0030493

**Published:** 2012-01-24

**Authors:** Hui Lin, Liang Hu, Leilei Chen, Hong Yu, Qi Wang, Ping Chen, Xiao-Tong Hu, Xiu-Jun Cai, Xin-Yuan Guan

**Affiliations:** 1 Department of General Surgery, Sir RunRun Shaw Hospital, School of Medicine, Zhejiang University, Hangzhou, China; 2 Department of Clinical Oncology, State Key Laboratory for Liver Research, The University of Hong Kong, Hong Kong, Special Administrative Region, People's Republic of China; 3 Institute of Reproductive and Stem Cell Engineering, Central South University, Changsha, China; 4 National Engineering Research Center of Human Stem Cells, Changsha, China; 5 Biomedical Research Center, Sir Run Run Shaw Hospital, School of Medicine, Zhejiang University, Hangzhou, China; Instituto Nacional de Câncer, Brazil

## Abstract

**Background:**

The identification and characterization of cancer stem cells (CSCs) is imperative to understanding the mechanism of cancer pathogenesis. Growing evidence suggests that CSCs play critical roles in the development and progression of cancer. However, controversy exists as to whether CSCs arise from bone marrow-derived cells (BMDCs).

**Methodology and Principal Findings:**

In the present study, n-nitrosodiethylamine (DEN) was used to induce tumor formation in female mice that received bone marrow from male mice. Tumor formation was induced in 20/26 mice, including 12 liver tumors, 6 lung tumors, 1 bladder tumor and 1 nasopharyngeal tumor. Through comparison of fluorescence *in situ* hybridization (FISH) results in corresponding areas from serial tumor sections stained with H&E, we determined that BMDCs were recruited to both tumor tissue and normal surrounding tissue at a very low frequency (0.2–1% in tumors and 0–0.3% in normal tissues). However, approximately 3–70% of cells in the tissues surrounding the tumor were BMDCs, and the percentage of BMDCs was highly associated with the inflammatory status of the tissue. In the present study, no evidence was found to support the existence of fusion cells formed form BMDCs and tissue-specific stem cells.

**Conclusions:**

In summary, our data suggest that although BMDCs may contribute to tumor progression, they are unlike to contribute to tumor initiation.

## Introduction

A growing body of literature suggests that tumors originate from a small portion of cells, referred to as cancer stem cells (CSCs) or tumor initiating cells (TICs) because these cells bear stem cell-like features such as self-renewal and differentiation [1]. To date, CSCs have been demonstrated to exist in cancers of the hematopoietic system [2], breast [3], brain [4], prostate [5], gastric [6], lung [7], colon [8], and liver [9]. However, little is known regarding the origin of CSCs. One possible origin of CSCs is the bone marrow, as bone marrow derived cells (BMDCs) are frequently found in tumor tissues and BMDCs have the ability to differentiate into many different types of cells including mesenchymal cells, muscle cells and epithelial cells, including hepatic cells. Recently, our knowledge of the relationship between BMDCs and cancer progression has dramatically improved. One interesting aspect is that cancer cells actively recruit BMDCs to their own microenvironment. BMDCs in tumors are not only responsible for inflammation but also for tumor angiogenesis [10]. CD45-positive BMDCs are frequently found in tumor tissue, where they express vascular endothelial cell growth factor receptor-1 (VEGFR-1) [11], a key receptor of VEGF. In addition to inflammation, these CD45+/VEGFR1+ cells also contribute to tumor angiogenesis. Thus, evidence demonstrates that BMDCs provide a suitable microenvironment to facilitate cancer metastasis [12].

However, it is unclear whether cancer cells originate from BMDCs, and this hypothesis is frequently debated. One recent report found that after chronic *Helicobacter* infection, BMDCs accumulated in the gastric mucosa and eventually gave rise to gastric cancer [6]. Furthermore, additional studies suggested that oncogenic mutations of tissue stem cells or further differentiated cells might create a pool of self-renewing cells in which those mutations accumulated and finally resulted in cancer [4], [5]. In bone marrow transplantation models, it was demonstrated that BMDCs were unlikely to be the origin of liver cancer [13] and skin cancer [14]. To test whether cancer originates from BMDCs, the chemical carcinogen n-nitrosodiethylamine (DEN) was used to induce tumor development in mice following bone marrow transplantation. The bone marrow of female recipient mice was eradicated by irradiation and then reconstituted with bone marrow from normal male mice. The Y chromosome was used as marker to characterize the origin of the induced tumor cells. Twenty tumors, including 12 liver tumors, 6 lung tumors, 1 bladder tumor and 1 nasopharyngeal tumor, were successfully induced. Among these tumors, clonal expansion of Y-positive (Y+) cells was not observed. The number of Y+ cells in the tumors closely correlated with the number of infiltrating lymphocytes. We also found that most Y+ cells expressed both CD45 and VEGFR-1. Our data suggested that, at least in our animal model, BMDCs are not the origin of cancer stem cells, although they are related to tumor inflammation and may contribute to the formation of tumor neo-vessels.

## Results

### Detection of Mouse X- and Y-chromosomes by FISH

**Figure 1:**
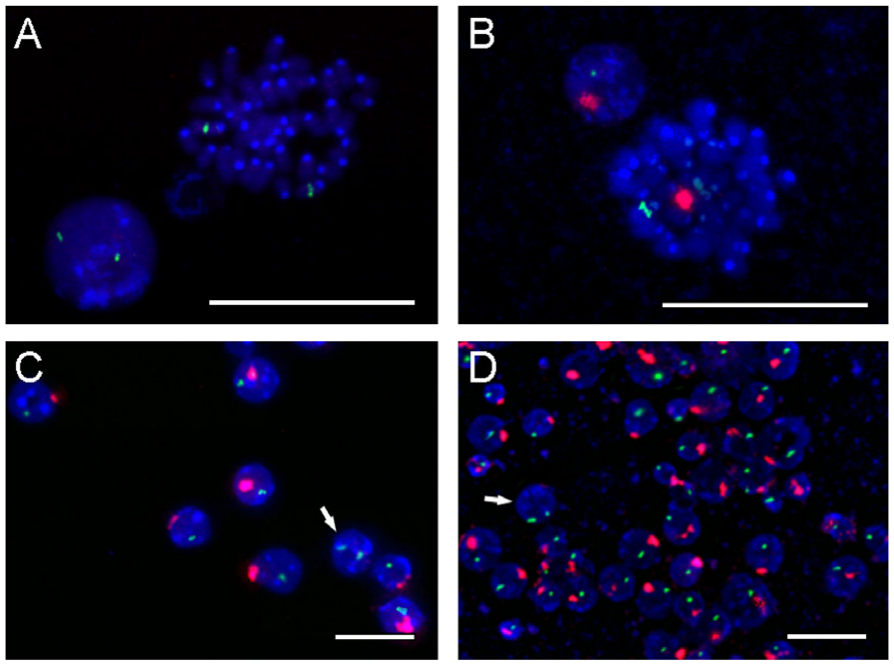
Representative FISH results using X (green) and Y (red) probes. (A) A metaphase peripheral blood lymphocyte from a female recipient mouse. (B) A metaphase peripheral blood lymphocyte from a male donor mouse. (C) Peripheral blood lymphocytes from a female recipient mouse after BMT. A female lymphocyte is indicated by an arrow. (D) Bone marrow cells from a female recipient mouse after BMT. A female bone marrow cell is indicated by an arrow. Magnification, ×100 (A and B) or ×40 (C and D). Scale bars, 20 µm.

For the bone marrow transplantation (BMT), bone marrow cells collected from 6 donor male mice were transplanted into 60 recipient female mice. As shown in Figure 1, FISH probes hybridized to both metaphase and interphase chromosomes and yielded strong and specific signals. FISH signals of the Y chromosome were only detected in cells from donor male mouse bone marrow (Figure 1B).

### Bone Marrow Transplantation

All recipient mice survived the BMT. The overall level of engraftment was 82.5–94.5% as assessed by calculating the percentage of Y-positive cells among both peripheral lymphocytes (Figure 1C) and bone marrow cells (Figure 1D). A total of 500 cells was analyzed in each sample. This result indicates that the BMT procedures were successful.

### DEN-Induced Carcinogenesis

**Figure 2:**
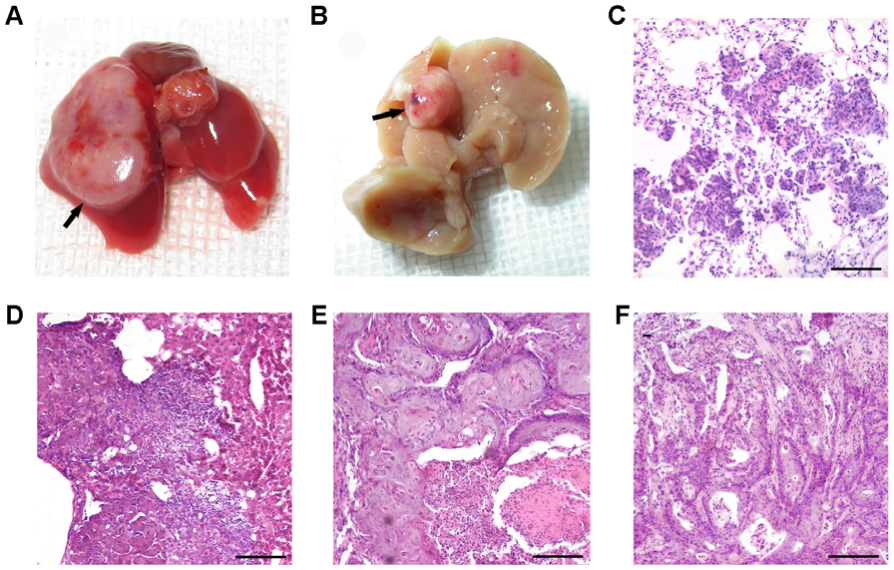
DEN-induced tumors in BMT mice. (A) Gross pathology of a representative lung cancer. (B) Gross pathology of a representative liver cancer. Representative H&E staining sections for adenocarcinoma of the lung (C), hepatocellular carcinoma (D), squamous cell carcinoma of the bladder (E), and nasopharyngeal cancer (F). Magnification, ×20 (C, D, E, and F). Scale bars, 50 µm.

During the 30 weeks of chemically induced carcinogenesis, 34 BMT mice died. Complete autopsies were performed on each of these mice, with the primary cause of death being extensive pneumonia and hepatic failure. Tumors were induced in 20 of the 26 surviving mice; these tumors included 12 liver tumors, 6 lung tumors, 1 bladder tumor and 1 nasopharyngeal tumor. Representative images of the lung and liver tumors are shown in Figure 2A and 2B, respectively. These tumors were resected, embedded in paraffin, sectioned and histologically characterized using H&E. Of the 6 induced lung cancers, 4 were characterized as squamous cell carcinoma, and the remaining 2 were characterized as adenocarcinoma (Figure 2C). All 12 liver tumors were characterized as hepatocellular carcinoma (Figure 2D). Both the bladder (Figure 2E) and nasopharyngeal cancers (Figure 2F) were characterized as squamous cell carcinoma.

### Distribution of BMDCs in DEN-Induced Tumors

**Figure 3:**
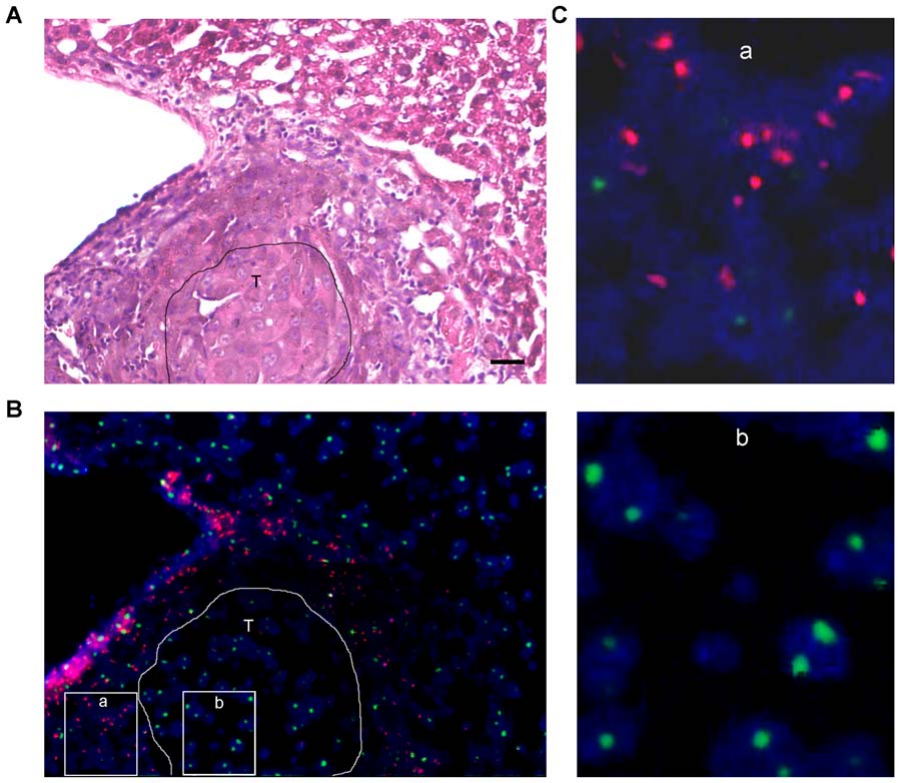
Comparison of the histopathological features and FISH data of adjacent serial sections of an HCC. (A) A representative H&E staining image demonstrating the histopathological features of the HCC tissue and the surrounding tissue. (B) A representative FISH image of the same area from an adjacent serial section constructed by the fusion of several images. (C) Magnification of areas from the tissue surrounding the tumor (a) and the tumor tissue (b) in (B). T, tumor tissue. Magnification, ×20 (A & B). Scale bars, 20 µm.

**Figure 4:**
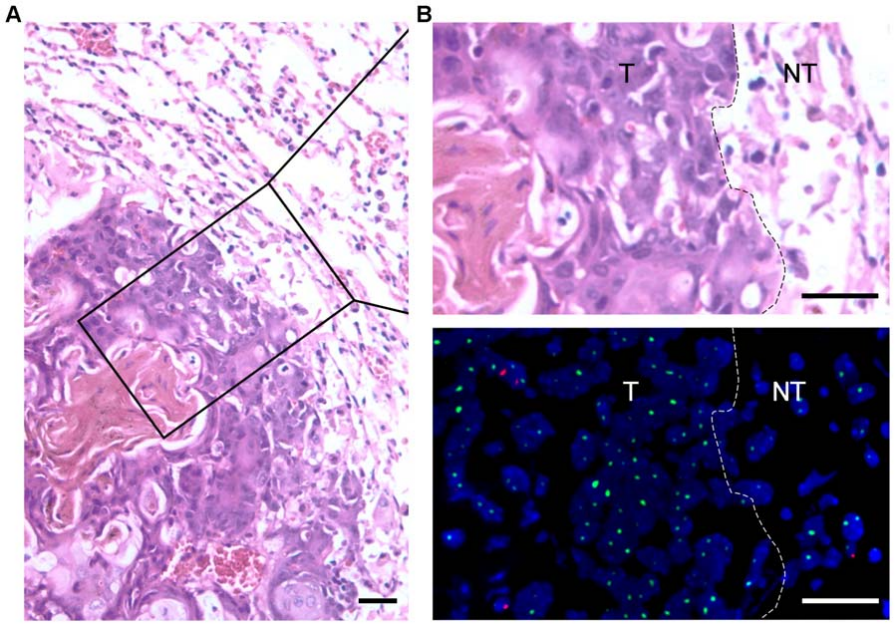
Comparison of the histopathological features and FISH data of adjacent serial sections of a squamous cell carcinoma in the lung. (A) H&E staining image presenting the histopathological features of the lung cancer and the surrounding normal tissue. Magnification, ×20. (B) H&E staining (upper) and FISH image (lower) of a magnified area in (A). T, tumor tissue; NT, non-tumor tissue. Magnification, ×40. Scale bars, 20 µm.

**Figure 5:**
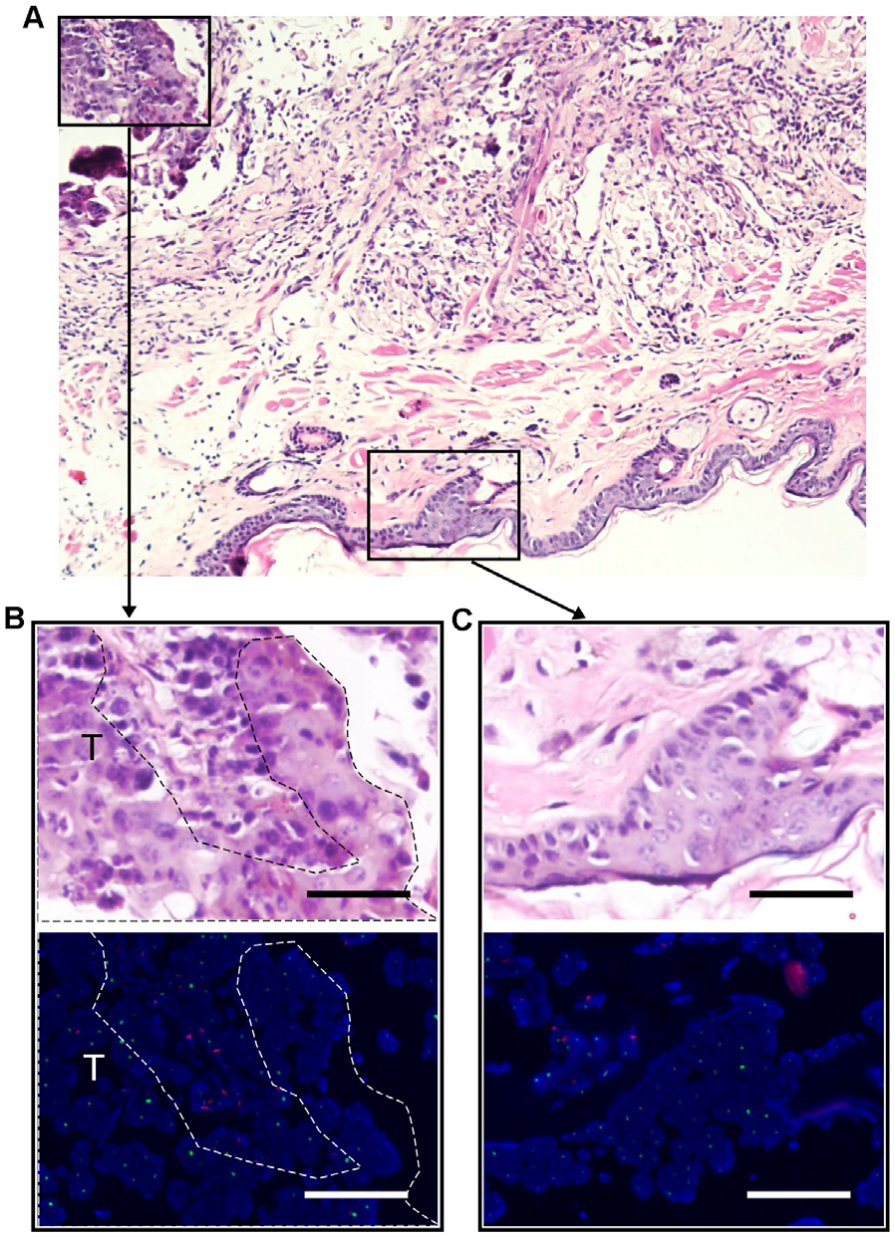
Comparison of the histopathological features and FISH data of adjacent serial sections of a nasopharyngeal carcinoma (NPC). (A) H&E staining image presenting the histopathological features of the NPC and the surrounding tissue. Magnification, ×20. (B & C) H&E staining (upper) and FISH image (lower) of the magnified areas in (A). T, tumor tissue. Magnification, ×40. Scale bars, 20 µm.

**Figure 6:**
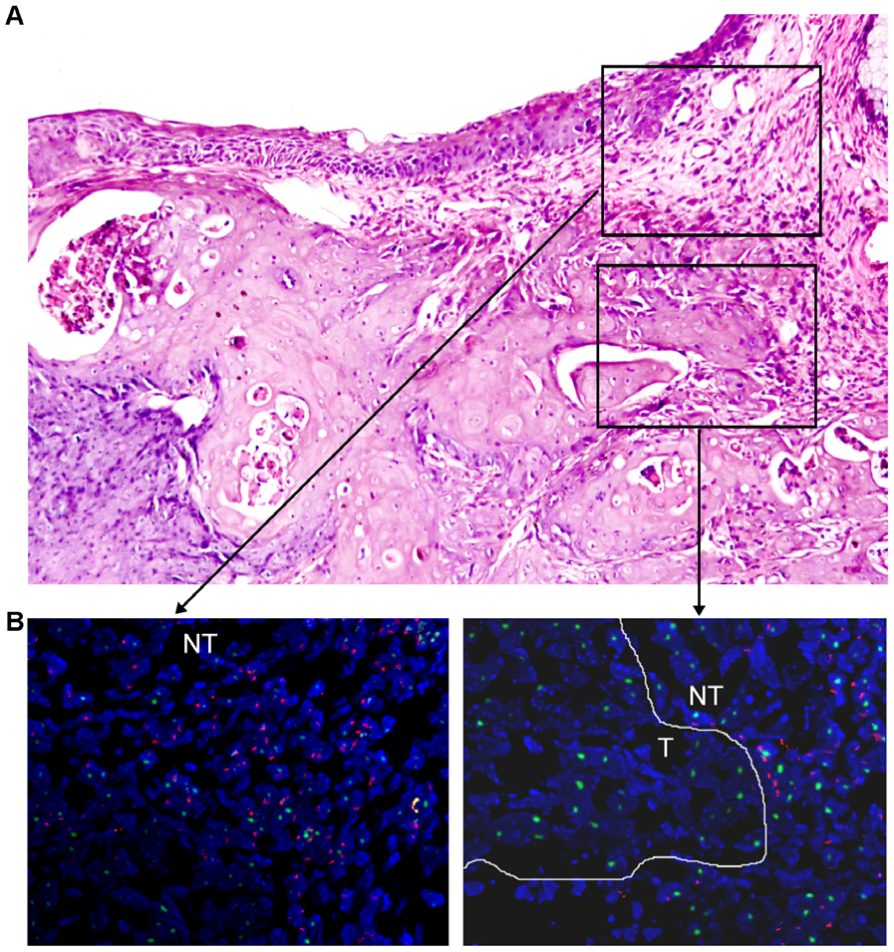
Comparison of the histopathological features and FISH data of adjacent serial sections of a bladder cancer. (A) H&E staining image presenting the histopathological features of the bladder cancer and the surrounding tissue. Magnification, ×20. (B) FISH images of magnified areas in (A). T, tumor tissue; NT, non-tumor tissue. Magnification, ×40.

To investigate whether DEN-induced cancers originated from bone marrow, we used FISH to determine the percentage of Y-positive (Y+) cells. In each case, 500–5,000 cells were counted to calculate the percentage of Y+ cells in tumor specimens. In all DEN-induced tumors, the frequency of Y+ cell was 0.2–0.6% in the liver cancers (Figure 3B and 3C), 0–0.3% in the lung cancers (Figure 4), 0.6% in the bladder cancer (Figure 5) and 1% in the nasopharyngeal cancer (Figure 6). Interestingly, the frequencies of Y+ cells were extremely high in the tissues surrounding the tumor, where a large percentage of infiltrating inflammatory cells and fibroblasts resided. The frequency of Y+ cells in the tissue surrounding the tumor was 30–50% in the liver tumors (Figure 3B, 3C), 3–8% in the lung tumors (Figure 4), 40% in the bladder tumor (Figure 5) and 70% in the nasopharyngeal tumor (Figure 6). However, a significant frequency of Y+ cells was not observed in the normal tissues adjacent to the liver, lung and bladder tumors. These data suggest that the cellular origin of the cancers is not the bone marrow.

To evaluate whether BMDCs can fuse with tumor cells, we analyzed the number of X and Y signals in BMDCs in the tumors and their surrounding tissues. No fusion-derived karyotype (XXY, XXXY) cells were found in our study. This result is consistent with many other findings [6], [11], [15], [16] and suggests that cell fusion is not the main contribution of BMDCs to malignant pathogenesis.

## Discussion

Although the cancer stem cell hypothesis remains debated, many scientists believe that cancers contain a small number of cancer stem cells (CSCs). These CSCs are able to self-renew, differentiate, and resist chemotherapy and thus maintain the cancer growth. To date, the origin of CSCs remains a mystery. At least three possible origins of CSCs have been proposed, including 1) tissue-specific stem cells [17]; 2) BMDCs [6], [15]; and 3) fusion cells derived from BMDCs and tissue-specific progenitor or stem cells [18]–[20]. In this study, we utilized a bone marrow transplantation animal model to investigate the origin of CSCs in chemically induced (DEN) tumors. DEN is a carcinogen that can induce tumors in various organs including the liver and skin and in the gastrointestinal, respiratory and hematopoietic systems in mice. When metabolized, DEN in converted into an electrophilic ethyldiazonium ion that can react with DNA bases and form adducts and thus may cause DNA mutations and strand breaks that ultimately contribute to pathogenesis [21]. In our model, we used a high dose of DEN (60 ppm) in drinking water for 12 weeks to increase the frequency and shorten the period of tumor formation. Using this strategy, tumors were successfully induced in the liver, lung, bladder and nasopharynx.

BMDCs have the ability to differentiate into epithelial cells during tissue repair in many organs, including the liver [22], [23], lungs, skin, eyes, GI tract and kidneys [24]. However, whether and how BMDCs account for tissue repair is still debated. Although the number of BMDCs was variable, most publications show that BMDCs were recruited to tissue damage sites [25]–[28], whereas other publication report that BMDCs were not detected in the models used [29], [30]. In murine liver and pancreatic tumor models, the more advanced tumors had a higher tendency to recruit BMDCs [31]. In our model, we found that the percentages of BMDCs in both tumor and normal tissues were very low (<1%); however, BMDCs were found in the tissue surrounding the tumor. This area is infilitrated with inflammatory cells, suggesting that these BMDCs were associated with inflammation. This result suggests that BMDCs were less likely to be transformed into malignant cells as none of the tumors in our model contained clustered Y+ cells.

Recent findings showed that BMDCs can incorporate into livers and intestines through cell fusion [18]–[20], which raised the possibility that BMDCs may play a role in regeneration and tumorigenesis by fusing with progenitor or stem cells of the target organ. However, many reports argue that there is no clear evidence to demonstrate that cell fusion occurs between BMDCs and target organ cells in their models [6], [11], [15], [16]. In the present study, we confirmed the lack of evidence to demonstrate cell fusion both in tumors and in surrounding normal tissue by FISH. Notably, although BMDCs were found incorporated into the intestinal adenoma in APC-mutated multiple intestinal neoplasia mice, none of adenomas ubiquitously consisted of BMDCs [20], suggesting that the mechanism by which BMDCs contribute to pathogenesis was not likely to be cell fusion. In summary, our data showed that although BMDCs may contribute to tumor progression, the possibility that BMDCs are the origin of the tumor itself is extremely low.

## Materials and Methods

### Bone Marrow Transplantation (BMT)

Six- to eight-week old male and female C57BL/6J mice were purchased from the Model Animal Research Center of Nanjing University (Nanjing, China). The animal experiments were performed in accordance with institutional guidelines, and the study was approved by the ethics committee of Zhejiang University (Approval ID: SYXK2004-0050). For the BMT, total bone marrow was isolated from the femurs and tibias of donor male mice, washed, triturated using a 20-gauge needle and passed through a 40-µm nylon mesh cell strainer (Becton Dickson, Franklin Lakes, NJ) to produce a single-cell suspension in PBS at a density of 1.5×10^7^ cells/ml. The recipient female C57BL/6J mice were irradiated with 900 rads from a ^60^Co irradiator, reconstituted with 3×10^6^ donor marrow cells via tail vein injection or retro-orbital sampling of anesthetized mice 36 hours later, and used for experiments 4–5 weeks after recovery. To confirm successful transplantation of donor bone marrow cells, recipient peripheral blood and bone marrow cells were obtained 4 weeks after the BMT and examined for the presence of the Y-chromosome with fluorescence *in situ* hybridization (FISH).

### Chemically Induced Carcinogenesis with n-Nitrosodiethylamine (DEN)

Approximately 4–5 weeks after BMT, 60 recipient mice were fed DEN (60 ppm in drinking water, Sigma, St Louis, MO) for 12 weeks for two sessions with a 2-week separation interval to initiate the process of carcinogenesis. The mice that survived were sacrificed at 30 weeks.

### Histological and Pathological Study

DEN-induced tumors were removed and fixed in 4% formalin overnight. After dehydration, the tumor tissue was embedded in paraffin. The tumor tissue in paraffin was serially sectioned (5 µm in thickness), and select slides were routinely stained with hematoxylin-eosin (H&E). All of the tumor sections were reviewed in a blinded manner by two experienced pathologists.

### Fluorescence *in situ* Hybridization

Two bacterial artificial chromosome (BAC) clones (RP24-0090J19 and RP24-185C22) containing the sry gene of the mouse Y chromosome were selected to identify cells derived from the male mice. The BACs containing the DNA fragment from the murine X7A.1 region (RP23-0170B5 and RP23-0011C16) were used as control probes for the murine X chromosome. Paraffin-embedded tissue sections adjacent to the H&E-stained sections were selected for FISH detection. Sections were deparaffinized, digested with protease K, and then hybridized with the fluorophore-labeled BAC probes according to the method described previously [32]. The FISH results were observed and captured using a fluorescence microscope equipped with a cooled CCD camera. The FISH signals were compared with the H&E staining of the same area in a successive tumor section.
